# How to become a successful invasive tapeworm: a case study of abandoned sexuality and exceptional chromosome diversification in the triploid carp parasite *Atractolytocestus huronensis* Anthony, 1958 (Caryophyllidea: Lytocestidae)

**DOI:** 10.1186/s13071-019-3420-0

**Published:** 2019-04-11

**Authors:** Marta Špakulová, Marta Bombarová, Dana Miklisová, Stanislava Nechybová, Iva Langrová

**Affiliations:** 1 Department of Zoology and Fisheries, Faculty of Agrobiology, Food and Natural Resources, Czech University of Life Sciences Prague, Kamýcká 129, 16500 Praha Suchdol, Czech Republic; 2 Institute of Parasitology, Slovak Academy of Sciences, Hlinkova 3, 04001 Košice, Slovakia; 3 Department of Genetics, Medirex Laboratories, a.s., Magnezitárska 2/C, 04013 Košice, Slovakia

**Keywords:** Cestoda, Polyploidy, Diversification of homologues, Interstitial telomere sequences (ITSs), Aberrant meiosis

## Abstract

**Background:**

A cytogenetic analysis of the new local triploid population of the caryophyllidean tapeworm *Atractolytocestus huronensis*, a unique parthenogenetic species with the ability to colonise new regions, was performed to understand the inner structure of its chromosome complement.

**Methods:**

A karyotype analysis was carried out using classical Giemsa staining and C-banding combined with fluorescent DAPI staining. A hypothesis that triplets are composed from three homologue chromosomes of approximately the same length and same centromere position was tested statistically for multiple dependent variables using a non-parametric Friedman’s ANOVA. The chromosomal location of ribosomal DNA clusters within the nucleolar organization region (NORs) and telomeric (TTAGGG)_n_ sequences were detected by fluorescent *in situ* hybridization (FISH). Chromosomes were subjected to AgNO_3_ staining in order to determine whether the rDNA sites represent active NORs.

**Results:**

The cytogenetic analysis confirmed the karyotype composed from eight chromosome triplets (3n = 24) as well as the existence of a pair of NORs located on each chromosome of the second triplet. Six NORs varied their activity from cell to cell, and it was reflected in the numbers of nucleoli (from 1 to 5). A huge morphological diversification of homologue chromosomes was originally detected in six out of eight triplets; the homologue elements differed significantly either in length and/or morphology, and some of them carried discernible interstitial telomeric sequences (ITSs), while the end telomeres were minute. The heterochromatin bands with high AT content varied irregularly, and the course of aberrant spermatogenesis was evident.

**Conclusions:**

Diversification of homologues is a unique phenomenon very likely caused by the long-term absence of a recombination and consequential accumulation of chromosome rearrangements in the genome of *A. huronensis* during species evolution. Unalterable asexual reproduction of the tapeworm, along with international trade in its host (carp), is facilitating its ongoing spread.

## Background

Caryophyllidean cestodes represent a unique group of tapeworms with respect to their morphology, host relations and evolutionary status [1, 2]. They have a monozoic body plan, tubificid annelids as their exclusive intermediate hosts, and cypriniform or siluriform fish as final hosts. Their basal or near-basal position in the cestode phylogeny has been resolved and repeatedly confirmed [3–5].

*Atractolytocestus huronensis* Anthony, 1958 (Caryophyllidea, Lytocestidae), is a widely distributed specific parasite of carp (*Cyprinus carpio* Linnaeus, 1758). The tapeworm was originally recorded from carp in the Huron River, Michigan, USA [6], and has been reported from feral and farmed carp in Asia, Europe, South Africa and China; this wide expansion was undoubtedly enhanced by the intensive fish trade [7–9]. This unique parthenogenetic species is well known genetically by its triploid nature, intra-individual ribosomal polymorphism, multiple rDNA loci [1, 10], and its stable triploidy accompanied by parthenogenetic reproduction [1, 10, 11]. In previous studies, it was shown that the chromosome complements of both European and American tapeworms comprised eight chromosome triplets, and their morphologies and classifications were similar. Moreover, both triploid *A. huronensis* populations had aberrant spermatogenesis as shown by light microscope drawings [1] and ultrastructure photomicrographs [12]. Due to a lack of functional sperm, it was concluded that parthenogenesis was the regular mode of reproduction of the species [1]. This tapeworm may thus be considered a stable, cosmopolitan and genetically and taxonomically unique species, as Jones & Mackiewicz [1] predicted.

The latest molecular analysis involving worldwide populations of *A. huronensis* [9], using mitochondrial *cox*1 sequences, indicated that the monophyletic species comprises two slightly differentiated lineages: one is better supported and involves tapeworms from China, the USA and the UK, while the other is represented by worms from continental Europe and South Africa. Using a haplotype network analysis, this study also indicated that the greatest population diversity of *A. huronensis* could be found in China, and therefore the eastern Palaearctic may be the location of the source population for global expansion of this invasive tapeworm.

This present study significantly expands knowledge concerning the detailed morphology of mitotic chromosomes, the activity and location of ribosomal DNA loci, distribution of telomeric repeats, and an aberrant course of meiotic spermatocyte development, using a series of cytogenetic approaches. Statistical evaluation of homologue chromosomes of individual triplets was used for the first time to infer chromosomal dissimilarities in the genome of *A. huronensis*. These new cytogenetic data, together with recent molecular analyses, allow us to discuss the polyploid origin of *A. huronensis*.

## Methods

### Parasites

Eight adult tapeworms of *A. huronensis* were dissected from the intestine of three common carp (*Cyprinus carpio carpio* L.) from an eight-hectare pond near Pozdišovce (eastern Slovakia, 48°44′00″N, 21°51′00″E). The pond was built in 1987 and it is currently used for recreational carp fishing. Fed by several forest streams, including the small Lipovec River, its water is considered unpolluted. The cestodes were identified using morphological species-specific characters from Oros et al. [13].

### Chromosome preparations

Live specimens of *A. huronensis* were processed immediately after carp dissection and incubated in a 0.025% colchicine saline solution for 1 h at room temperature. The tapeworms were then incubated in a hypotonic solution of 0.6% sodium citrate for 12 h at 4 °C. Thereafter, the tegument of each tapeworm was torn gently along the body using needles (in order to facilitate penetration of fluid into the worm tissues) and the worms were then placed into a freshly prepared cold fixative solution (3:1 methanol:acetic acid) for 2 h (with two replacements of fixative). The material was stored at -20 °C until needed.

Chromosome spread preparations were made according to Frydrychová & Marec [14]. Pieces of unfrozen worms containing testes and vitelline glands were transferred into a drop of 60% acetic acid on a clean slide and torn into fine pieces using tungsten needles. The slide was then placed on a heating plate at 45 °C and the drop was slowly drawn along the slide until the liquid evaporated. Preparations were either stained with a 5% solution of Giemsa (Merck, Kenilworth, NJ, USA) in a phosphate buffer (pH 6.8) for 30 min and rinsed with tap water, or dehydrated in an ethanol series (70, 80 and 100%, 30 s each) and stored at -20 °C until further use.

### Karyological and statistical analyses

The best 14 mitotic spreads were selected for detailed analysis of the length and morphology of individual chromosomes. The chromosome set of each cell was sorted into triplets according to their length; however, the presence of ribosomal loci on triplet number 2 was also considered. The length of chromosome arms was measured and the centromeric index was calculated. The classification of chromosomes as metacentric, submetacentric and acrocentric was used [15]; this system, often used in mammalian and human cytogenetics, allows an uncomplicated comparison of basic chromosome morphology, which was one of the work goals. Statistical analyses were performed using the Excel (Microsoft Office 2007) and STATISTICA v.12.0 software packages (StatSoft, Inc. 2013). A hypothesis that the triplets are composed from three homologue chromosomes of approximately the same length and same centromere position was statistically tested for multiple dependent variables using a non-parametric Friedman’s ANOVA. Each test for triplets 1 through 7 was followed by a *post-hoc* test in order to precisely determine which neighbouring chromosome pairs were responsible for the statistical significance of the differences. All tests were two-tailed with a significance probability designation of *P* = 0.05.

### C-banding and DAPI staining (AT-rich bands)

C-bands (AT-rich bands) were induced through a modification of a technique used by Fernández et al. [16], which basically involves heat denaturation of chromosomal DNA in the presence of formamide, followed by incubation in 2× SSC (saline-sodium citrate buffer) at room temperature. Chromosome slides were dehydrated for 30 s in an ethanol series of 70, 80 and 98%, and kept at 65 °C for 48 h before further use. Each slide was then coated with 20 µl of 50% formamide in 2× SSC, enclosed with a cover slip, denatured for 2 min at 70 °C, and incubated at 37 °C for 1 h. After incubation, the slide was rinsed in 2× SSC for 30 to 60 min at room temperature.

The chromosome preparation was then stained with 0.5 µg/ml DAPI (4′,6-diamino-2-phenylindole; Sigma-Aldrich, Gillingham, UK) in PBS containing 1% Triton X-100 for 5 min, washed at room temperature in 1% Kodak-PhotoFlo (Kodak Alaris Inc., Rochester, NY, USA) in PBS and in 1% Kodak-PhotoFlo in miliQ water for 4 and 1 min, respectively. Finally, the slides were mounted in 25 µl of antifade based on DABCO (1,4-iazabicyclo[2.2.2.]octane; Sigma-Aldrich, Gillingham, UK).

### Silver nitrate (Ag-NOR) staining

Silver nitrate stains the nucleolar organization region (NOR)-associated protein, producing a dark band wherein the silver is deposited; this method denotes the activity of ribosomal RNA genes within the NOR. Staining was performed according to Ráb & Roth [17]: two drops of water solution containing 50% AgNO_3_ and one drop of 2% gelatine in 1% formic acid were mixed on the surface of a cover slip. The slip was turned and attached on the chromosome preparation, and the slide was incubated on a heating plate at 45 °C until the colour changed to light brown. Then, the chromosomes were checked and photographed using a light microscope.

### Fluorescent *in situ* hybridization (FISH) with *18S* rDNA and telomeric probes

FISH is a technique that uses fluorescent probes binding selectively the chromosome parts with a high degree of sequence complementarity, thus detecting and localizing the presence or absence of specific DNA sequences on chromosomes. In the case of *A. huronensis*, *18S* rDNA and telomere probes were applied as follows.

The probe *18S* rDNA was generated by PCR with *18S* rDNA primers (18S-WormA forward 5′-GCG AAT GGC TCA TTA AAT CAG-3′ and 18S-WormB reverse 5′-CTT GTT ACG ACT TTT ACT TCC-3′; the dNTP mix contained 0.35 mM biotin-16-dUTP (Roche Diagnostics, Rotkreuz, Switzerland).

The ancestral (TTAGGG)_n_ sequence of the telomeric DNA was tentatively applied in *A. huronensis*. The probe was generated by non-template PCR using two primers, vertebrate-like (TTAGGG)_n_ forward (5′-TTA GGG TTA GGG TTA GGG TT-3′) and vertebrate-like (TTAGGG)_n_ reverse (5′-AAC CCT AAC CCT AAC CCT AA-3′) [18]. The probe was labelled by nick translation with biotin-14-dATP using BioNick Labeling System (Invitrogen Life Technologies, Carlsbad, CA, USA) at 16 °C for 1 h.

For FISH, the procedure described by Fuková et al. [19] was used. In particular, chromosome preparations were digested with 100 µg/ml RNase A in 2× SSC for 1 h at 37 °C and washed twice in 2× SSC for 5 min each. The slides were incubated in 5× Denhard’s solution for 30 min at 37 °C. Denaturation of chromosomal DNA was done in 70% formamide in 2× SSC for 3 min and 30 s at 68 °C. The probe cocktail per slide contained ~25 ng of biotinylated probe and 25 µg of sonicated salmon sperm DNA (Sigma-Aldrich, St. Louis, MO, USA). Post-hybridization washes consisted of 3 × 5 minutes in 50% formamide (Fluka, Buchs, Switzerland) in 2× SSC at 46 °C, 5 × 2 min in 2× SSC and 3 × 5 min in 0.1× SSC at 62 °C, and 3 × 3 min in 4× SSC containing 0.1% Tween 20 at 37 °C. Hybridization signals were detected with Cy3-conjugated streptavidin (Jackson ImmunoResearch Laboratories, West Grove, PA, USA), amplified with one round of biotinylated anti-streptavidin (Vector Laboratories Inc., Burlingame, CA, USA) and Cy3-conjugated streptavidin. The preparations were counterstained with 0.5 µg/ml DAPI and mounted in DABCO.

### Microscopy and image processing

Unstained preparations were screened under phase contrast microscopy using a Leica DM 1000 LED (Leica Microsystems, Wetzlar, Germany). Stained slides were inspected under a light and fluorescence microscope (Leica DM 4000 B) and dividing cells were photographed using a digital camera (DFC 450 C; Leica Microsystems, Wetzlar, Germany). For construction of the karyotype, microphotographs were processed by Adobe Photoshop, v.11.0.

## Results

### Mitotic karyotype

**Fig. 1:**
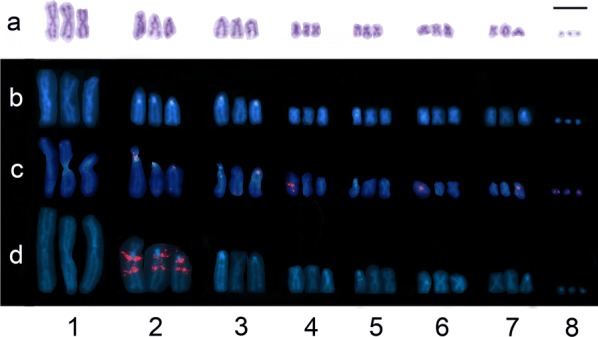
Karyotype derived from mitotic cells of *Atractolytocestus huronensis*. **a** Giemsa staining. **b** C-banded chromosomes showing AT-rich bands (labelled in light blue), counterstained with DAPI (blue). **c** FISH with telomeric probe (TTAGGG)_n_ (red) counterstained with DAPI (blue); Interstitial telomeric signals are located on of the chromosomes of triplets nos 2, 4, 6, 7 and 8. **d** FISH with *18S* rDNA probe (red) counterstained with DAPI (blue); two loci for *18S* rDNA (NORs) are located on each chromosome of the triplet no. 2. *Scale-bar*: 10 µm

**Fig. 2:**
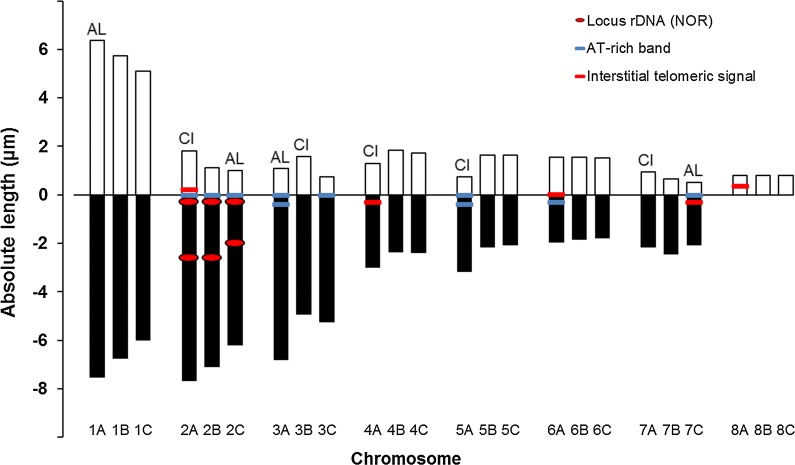
Ideogram for *Atractolytocestus huronensis* with marked ribosomal loci, AT-rich bands and interstitial telomeric signals. *Abbreviations*: AL, chromosomes significantly differing from the rest homologues in absolute length; CI, chromosomes differing in centromeric index (*post-hoc* test with significance level 0.05)

**Fig. 3:**
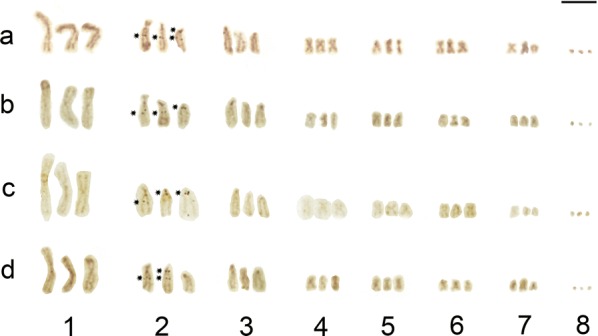
Karyotype derived from Ag-NOR stained mitotic cells of *Atractolytocestus huronensis*. Four (**a**) and three (**b-d**) active NORs randomly located on homologue chromosomes of the pair no. 2. *Scale-bar*: 10 µm

The chromosome number was determined from 105 mitotic metaphase complements isolated from tissue containing multiple testes of eight tapeworm individuals. Out of the cells, 98.6% possessed a complete number of 24 chromosomes comprising eight triplets (3n = 24, *n* = 8; Figs. 1, 2, 3); the rest were aneuploid without one, two or three smaller chromosomes. Despite an assumption that triplets are composed from homologue chromosomes of approximately the same length and the same centromere position, conspicuous differences were noticed within nearly all triplets except of the shortest triplet 8; it comprised 3 minute elements without a visible centromere (Fig. 1). Therefore, individual chromosomes were sorted by length and each of them was designated according to the triplet number and the order within the triplet (e.g. 1A, 1B, 1C; Fig. 2). The mean absolute length, centromere index, classification of individual elements, and presence of banding patterns are summarised in Table 1.

**Table 1:** Measurements and intra-triplet comparison of individual chromosomes of *Atratolytocestus huronensis* (*n* = 14)

Triplet no.	Chromosome	Absolute length (µm)	Centromeric index	Classificationª	Note^b^
1	A	13.9 ± 2.2*	45.8 ± 2.2	m	
	B	12.5 ± 1.6	45.9 ± 2.6	m	
	C	11.1 ± 2.1	46.0 ± 1.7	m	
2	A	9.5 ± 1.9	19.2 ± 2.4*	a	intTS, AT-rb, 2×NOR,
	B	8.2 ± 1.6	13.6 ± 2.8	a	AT-rb, 2×NOR
	C	7.2 ± 1.4*	12.6 ± 2.5	a	AT-rb, 2×NOR
3	A	7.9 ± 1.4*	13.7 ± 2.4	a	2×AT-rb
	B	6.5 ± 1.2	24.2 ± 3.1*	a-sm	
	C	6.0 ± 1.1	12.5 ± 3.5	a	AT-rb
4	A	4.3 ± 0.7	30.3 ± 7.4*	sm	intTS
	B	4.2 ± 0.7	43.5 ± 2.7	m	
	C	4.1 ± 0.6	42.0 ± 3.8	m	
5	A	3.9 ± 0.6	19.0 ± 1.9*	a	2×AT-rb
	B	3.8 ± 0.5	43.4 ± 3.6	m	
	C	3.7 ± 0.7	44.1 ± 3.1	m	
6	A	3.5 ± 0.6	44.4 ± 3.3	m	intTS, AT-rb
	B	3.4 ± 0.6	46.0 ± 2.5	m	
	C	3.3 ± 0.5	45.9 ± 3.0	m	
7	A	3.1 ± 0.6	30.5 ± 6.2*	sm	
	B	3.1 ± 0.6	21.4 ± 4.0	a	
	C	2.6 ± 0.5*	20.0 ± 3.5	a	AT-rb, intTS
8	A, B, C	0.8 ± 0.3	–	–	

^a^Classification according to Dos Santos Guerra [15]: m, metacentric; sm, submetacentric; a, acrocentric chromosome pair

^b^intTS, interstitial telomeric signal; AT-rb, AT-rich band; NOR, cluster of ribosomal DNA

*Significant difference (*P* < 0.05) from the neighbour chromosome of the same triplet

Using Friedman’s ANOVA for multiple dependent variables, the non-parametric association among the chromosome length of each triplet members was tested, and the results of the ANOVA (*n* = 14, *df* = 2) with exact *P-*values for dependent variables are shown in Table 2.

**Table 2:** Test of differences among chromosomes of individual triplets (nos 1–7) of *Atractolytocestus huronensis* karyotype

Triplet no.	Absolute length		Centromeric index	
	ANOVA *χ*^2^	*P*	ANOVA *χ*^2^	*P*
1	27.11	0.0001*	1.00	0.606
2	25.76	0.0001*	20.76	0.0001*
3	21.78	0.0001*	21.14	0.0001*
4	6.95	0.031*	21.57	0.0001*
5	21.42	0.0001*	21.42	0.0001*
6	6.65	0.036*	0.94	0.624
7	13.33	0.0001*	20.76	0.0001*

*Significant difference *P* < 0.05; critical value for (*n* = 14, *df* = 2) = 5.99

Generally, all triplets 1 through 7 showed significant differences for the chromosome length (*P* < 0.05); however, chromosomes of triplets 4 and 6 were most similar to each other showing limit values. The same statistical evaluation was made for the centromeric index and significant differences were shown for triplets 2, 3, 4, 5 and 7 (Table 2).

The *post-hoc* test showed specifically that the chromosome 1A was longer than two remaining triplet members 1B and 1C (Table 1). The length of 1B and 1C differed in some cells but the test was insignificant. The morphology of all three chromosomes of the first triplet was metacentric and no difference was revealed in centromeric indices.

Regarding triplet 2, the first chromosome, 2A, differed in a higher centromeric index, having clear short arms (see Figs. 1, 2, 3), and chromosome 2C was significantly shorter than 2A and 2B (Figs. 1, 2, 3, Table 1).

Within the third triplet, chromosome 3A was the longest and 3B had longer short arms, being on the border between acrocentric and submetacentric classification (Table 1).

No significant difference was determined by the *post-hoc* test for the length of chromosomes 4A, 4B and 4C, in spite of the limit result of the initial ANOVA *χ*^2^. However, chromosome 4A differed in centromeric index, being classified as submetacentric while the remaining homologue pair was metacentric.

Similar results were found for the fifth triplet. The length of chromosomes slightly decreased but a significant difference was found only between 5A and 5C. Chromosome 5A also differed in the location of centromere being acrocentric, while 5B and 5C were metacentric.

The sixth triplet comprised short metacentric chromosomes which differed insignificantly in length and centromere location, in spite of the limit result of the initial ANOVA test (see Table 2).

The seventh triplet also contained specific chromosomes. Chromosome 7A was submetacentric, having longer short arms than acrocentrics 7B and 7C. Moreover, 7C was significantly shorter in the majority of cells (Tables 1, 2; Figs. 1, 2, 3).

### Number and chromosomal location of ribosomal DNA clusters (NORs) and their activity

Fluorescent *in situ* hybridization with the *18S* rDNA probe revealed two loci of rDNA (i.e. nucleolar organization regions, NORs) located interstitially on the long arms of all three chromosomes of the triplet 2 (Figs. 1d, 2). One locus was post-centromeric (2q cen) while the second was clearly intercalar (2q int). A functional activity of the ribosomal genes was proved using the silver staining (Ag-NOR banding) which revealed an outstanding variability of positively stained loci, most often being 3–4 in number on the homologues of the second triplet (Fig. 3). The layout of active ribosomal loci was random but the most frequent active locus was the intercalar one on chromosome 2A (2Aq int) (Fig. 3a-d). The number of nucleoli varied in range from 1 to 5 per interphase cell nucleus (not shown).

### Number and chromosomal location of AT-rich bands and interstitial telomeric sequences (ITSs)

Remarkable variation was detected within the triplets in number and chromosome location of both AT-rich blocks and interstitial signals of telomeric DNA (Figs. 1, 2).

The C-banded and DAPI-stained AT-rich blocks of heterochromatin appeared on three, two, or only a single chromosome of triplets 2, 3, 5, 6 and 7 (Fig. 1b, 2). Each homologue of triplet 2 carried the block located on the long arm very close to the centromere (2q cen); chromosomes 3A and 5A had a doubled block on the long arm behind the centromere, and chromosomes 3C, 6A and 7C each showed a single post-centromeric AT-rich block.

The interstitial telomeric signals (ITSs) were found on one member of triplets 2, 4, 6, 7 and 8 (Fig. 1c, 2). Chromosomes 2A and 6A had a pre-centromeric or centromeric block, while chromosomes 4A and 7C showed the telomeric signal close behind the centromere. The signal visible on the minute 8A chromosome could not be localized further. The regular telomeres located on chromosome ends were stained but the signals were extremely weak (Fig. 1c).

### Meiotic division

**Fig. 4:**
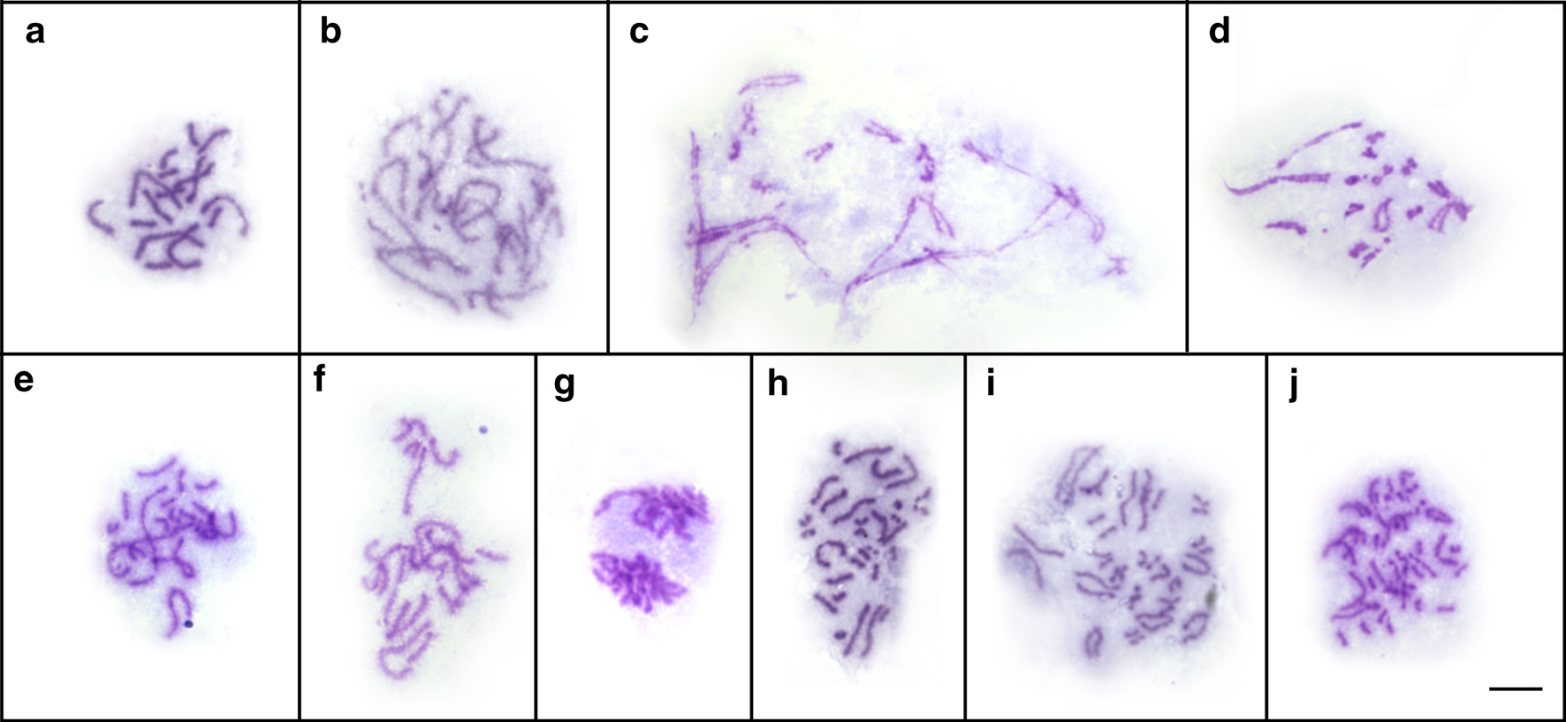
Aberrant meiotic division of spermatocytes of *Atractolytocestus huronensis*. Giemsa staining. **a** Pachytene with bivalents and univalents. **b**, **c** Early (**b**) and late (**c**) diplotene showing a synapsed trivalent, associations of bivalents, and several univalents. **d**, **e** Early (**d**) and late (**e**) diakinesis showing a trivalent, bivalents and univalents in each cell. **f** Late metaphase-early anaphase I; early segregation of univalent chromosomes to opposite poles while bivalents dwell in the cell centre. **g** Late anaphase I. **h** Late metaphase II. **i** Early anaphase II with splitting chromatids. **j** Early telophase II: a cluster of four aberrant spermatid nuclei. *Scale-bar*: 10 µm

**Fig. 5:**
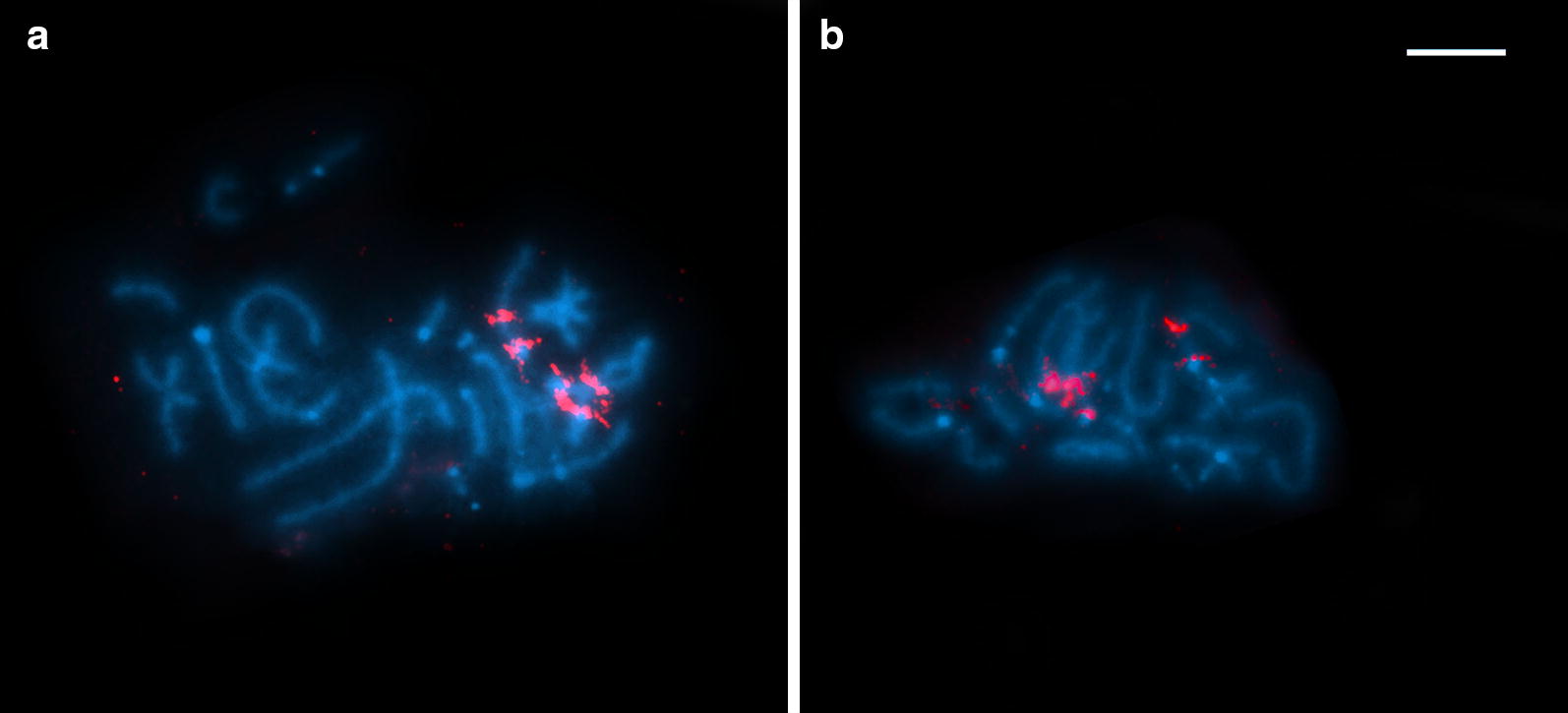
Irregural prophase pairing of spermatocyte chromosomes of *Atractolytocestus huronensis*. FISH with *18S* rDNA probe (red) counterstained with DAPI (blue). **a**, **b** Pachytene with NOR-bearing triplet in form of one bivalent and one univalent (**a**), or three univalents (**b**). *Scale-bar*: 10 µm

Selected meiotic stages of the aberrant course of spermatocyte division are shown in Figs. 4 and 5. Both the heterotypic and homeotypic divisions showed huge irregularities, such as variations in the chromosome number, laggard chromosomes, and unstable and anomalous configurations at diakinesis/metaphase I. During the meiotic prophase, chromosomes displayed several types of pairing association (univalents, bivalents and trivalents) (Figs. 4a-e, 5a, b). The shorter chromosomes remained mostly in univalent form (Fig. 4a-e) while the formation of bivalents and usually a trivalent was unbalanced and varied from cell to cell within an individual worm. An uncoordinated behaviour of prophase spermatocyte chromosomes was illustrated using fluorescent *in situ* hybridization with an rDNA probe (rDNA FISH) that marked a pair of ribosomal loci (red spots) localised on all three chromosomes of triplet 2 (Fig. 5). The homologues of this triplet formed either one bivalent plus one univalent (Fig. 5a), or they did not pair at all, remaining as three univalents (Fig. 5b). Subsequent stages of the heterotypic division (metaphase I, anaphase I; Fig. 4f, g) were not so frequent and the segregation of chromosomes to opposite cell poles was irregular, thus the remaining secondary spermatocyte cells contained a random chromosome number (Fig. 4g). The segregation of univalent chromosomes during early anaphase I often preceded the group of bivalents dwelling in the centre (Fig. 4f). Similarly, the segregation of chromatids during the following homeotypic division was irregular (Fig. 4h, i) leading to the formation of aneuploid gametes possessing unbalanced chromosome numbers (Fig. 4j).

## Discussion

The purpose of our study is to provide new cytogenetic data on *A. huronensis* and to improve previous karyological analyses of *A. huronensis* from the USA [1] and Europe [10, 11]. All previous studies clearly proved that the triploid tapeworm karyotype comprises 8 chromosome triplets (3n = 24). The slight discrepancy in classification of triplets 2, 3 and 7 (acrocentric *versus* subtelocentric) reported in geographically distant parasite populations (USA, Europe) might be based on either nomenclature differences or different karyological techniques used [1, 10]. Another view might relate to the above-mentioned affiliation of the American and European tapeworms to phylogenetically differentiated intraspecific clusters [9]. However, this last distinction was really insignificant as the total intrapopulation diversity was extremely low. In any case, chromosome characteristics should be re-examined in most world locations using standardized techniques.

Nevertheless, previous cytogenetic studies have not analysed morphological differences among homologue chromosomes within individual triplets, although photos from Králová-Hromadová et al. [10] suggest their possible existence. The analysis in the present study deals with newly collected worms of *A. huronensis*. These specimens were taken from a relatively isolated pond in eastern Slovakia, which is regularly stocked with carp. The cytogenetic analysis not only confirms the triploid nature of the *A. huronensis* karyotype but also reveals a much more intimate chromosome structure. Indeed, nearly all triplets were created from statistically distinct elements differing in macromorphology and/or banding patterns. As shown in Fig. 2, each of triplets 1, 4, 5 and 6 included a pair of slightly similar homologues (1BC, 4BC, 5BC, 6BC); however, even these similar elements within pairs might be more or less unequal in length. The third element of each mentioned triplet was visibly different, deviating significantly in length (1A), morphology (4A, 5A) or in the presence of AT-rich bands and/or interstitial telomeric signals (4A, 5A, 6A). The inner heterogeneity within triplets 2, 3 and 7 was even greater: each triplet possessed morphologically and structurally diverse chromosomes which differed in nearly all of the above-mentioned features (see Figs. 1, 2).

As with the previous analysis of another European population [10], two tandemly arranged clusters of ribosomal DNA (i.e. nucleolar organizer regions, NORs) were located on the long arm of chromosomes 2A, 2B and 2C. One cluster was found in the in the post-centromeric and the other in the intercalary position. However, silver-staining analysis, which highlights only active NORs [20], showed that not all the ribosomal gene loci were regularly functional, and the number of positive signals varied from cell to cell. This fact was reflected in the numbers of nucleoli (from one to five) apparent in interphase cells (not shown).

Successful application of the telomeric (TTAGGG)_n_ probe in *A. huronensis* has proved the existence of this ancestral sequence repeat in additional cestode species, which has been detected so far in two caryophyllidean and one nippotaeniidean tapeworms [21]. However, extremely weak terminal telomeres were accompanied by the irregular presence of interstitial telomeric sequences (ITSs) detected in five individual chromosomes, each belonging to various triplets. These interstitial signals, positioned close to the centromeres, had more intense colours in comparison to repeats on the chromosome ends. In general, ITSs, known in many eukaryotic genomes (from yeast to human), are thought to be linked to the disruption of genome integrity, but the detailed molecular mechanisms responsible for ITSs-mediated genome instability remain unclear [22, 23]. However, it has been proposed that ITSs are usually derived from ancestral telomere fusion events during karyotype evolution [24, 25]. They could act as hotspots for breakage and induce high rates of chromosome rearrangements; the breakage and fragility might facilitate chromosome remodelling and cell transformation [26]. In *A. huronensis*, ITSs occur only in one of three elements of triplets 2, 4, 6, 7 and 8. However, minimization of telomeres at the chromosome ends was evident in all elements.

The extreme diversification of chromosomes within individual triplets was evident in the course of the meiotic prophase of spermatocytes. Similarly to the previous analysis by Jones & Mackiewicz [1], a different frequency of univalents, bivalents and trivalents was detected depending on the type of individual chromosome. Our study clearly confirmed that meiotic division is abnormal and that spermatogenesis fails to produce functional sperm. While the course of spermatogonial mitoses seemed regular, all stages of spermatocyte divisions were aberrant. During the prophase stages, irregular multivalent associations or unpaired univalents were common. In the segregation of chromosomes to opposite poles during the early anaphase, univalents predated the paired multivalents which dwelled longer in the cell centre; as a result, this process was once again considered uneven. Homeotypical division results in abnormal sperm containing an irregular number of chromosomes. Using section methodology, Jones & Mackiewicz [1] described additional meiotic disorders such as chromosome non-disjunctions accompanied by spindle irregularities, as well as bridges between spermatid nuclei in telophase II (due to lagging chromosomes), etc. One ultramicroscopic study [12] showed a fragmentation of nuclei in *A. huronensis* spermatocytes, which is clearly a feature of cell degeneration and can be a consequence of the aberrant first meiotic division. That study did not detect any mature, functional spermatozoon [12]. In summary, all existing studies show that *A. huronensis* is a triploid parthenogen. It is, moreover, the only cestode species in which triploidy, parthenogenesis, multiple loci for ribosomal DNA (NORs) and intragenomic ITS paralogues are mutually linked [9, 10]. Such a combination of original genetic phenomena was also observed in a lung fluke [*Paragonimus westermani* (Kerbert, 1878), Digenea: Paragonimidae], a parasite that belongs to a species complex and has triploid populations occurring in sympatry with diploid and occasionally with tetraploid specimens in eastern Asia [27, 28].

Among tapeworms, the tendency for triploid individuals, or even triploid populations, to occur within a common diploid species is obvious in three basal groups of Eucestoda, namely the orders Spathebothriidea, Diphyllobothriidea and Caryophyllidea [29]. However, *A. huronensis* is the only stable, exclusively triploid parthenogenetic tapeworm species. Such cases are frequent among plants but extremely rare in animals. An example of a well-researched triploid invertebrate is the marbled crayfish [*Procambarus virginalis* (Lyko, 2017), Decapoda, Procambaridae] [30–32] which is an all-female species reproduced by cloning (apomictic parthenogenesis). *Atractolytocestus huronensis* and *P. virginalis* are the only obligatory parthenogens in their groups (Cestoda and Decapoda), and their type of reproduction is consistent with the extremely reduced diversity or even genetic identity of mitochondrial DNA [9, 30]. Both are successful invasive organisms with the potential to colonize new territories [33–35]. The crayfish has been distributed worldwide *via* the pet trade and has demonstrated its viability once released into the wild. The specific carp endoparasite *A. huronensis* is widespread together with its fish host throughout four continents [9]. However, through a series of phylogenetic approaches, it has been proven that the marbled crayfish is an evolutionarily young autopolyploid descendant of the sexually reproducing diploid congener, the slough crayfish (*P. fallax* Hagen, 1870) [32]. The origin of the polyploid nature of *A. huronensis*, however, is still uncertain.

Jones & Mackiewicz [1] took into account both possible variants of the hypothetic origin of the *A. huronensis* triploidy (autopolyploidization or interspecific hybridization). The authors expressed the view that if triploidy arose by genetic but not interspecific hybridization, “then the immediate ancestor of the triploid line may still exist in the carp” (p. 1117 in [1]). Two possible living candidates for ancestors may be the only two congeners *Atractolytocestus tenuicollis* (Li, 1964) and *A. sagittatus* (Kulakovskaya & Akhmerov, 1965), both of which parasitize the common carp exclusively [7, 36]. Both species appear to reproduce sexually, and their distribution is restricted to several regions in East Asia [7, 11]; *A. huronensis* is, on the contrary, a successful invasive species, widespread nearly worldwide, including the lower Chinese Yangtze River basin [9]. These data are consistent with the hypothesis that a non-overlapping spatial distribution pattern of sexuals and parthenogens is frequently found; typically, the sexual populations are located in the distribution centre while parthenogens are present at the margin of the distribution [37, 38]. Since all three *Atractolytocestus* congeners are specific carp parasites, it is likely that all of them were distributed worldwide; however, only the parthenogenetic *A. huronensis* possessed the ability to survive and complete its life-cycle out of Asia. In fact, parthenogenesis, together with polyploidy, of asexual lineages in new habitats are thought to be an ecological advantage when compared to their sexual diploid counterparts [33].

Recently, new light has been shed on certain problems concerning the molecular phylogeny of both the parasite *A. huronensis* [9] and its fish host *C. carpio* [39]. An analysis of various samples of *A. huronensis* from continental Europe, UK, USA, China and South Africa, along with the haplotype network based on ribosomal ITS2 variants (paralogues), reveals that there is a relatively increased level of diversity in tapeworm populations from China. This fact indicates that the region of East Asia likely played a role in the origin of *A. huronensis* and might serve as a source location for global expansion of this tapeworm. Molecular analysis of world carp populations [39] revealed that the latest round of genome duplication (allotetraploidization, 2n = 100) occurred approximately 8.2 million years ago, and a single origin of *C. carpio* from the Caspian Sea was confirmed. The previously designed [40, 41] historic distribution of carp into Europe and the eastern mainland of Asia is accepted as a very likely scenario. Moreover, carp domestication was confirmed through the Middle Ages; two independent regions were involved, namely the Roman empire and East Asia [39]. Since then, the long-lasting, intense worldwide carp trade was illustrated by extensive genetic admixtures occurring in both the feral and farmed *C. carpio* populations. The European lineage of mirror carp carried admixtures from Asian populations, whilst North American carp showed evidence of multiple introductions from both Europe and Asia [39].

Historically, the carp became a host of several sexual caryophyllidae parasites in Asia; however, the origin of triploidy in *A. huronensis* remains unknown. The current discrepancies within chromosome triplets suggest that a more likely explanation may be of hybrid origin, which is a major route to parthenogenesis in animals [33]. The triploid karyotype of *A. huronensis* comprises two more or less similar chromosome sets while the third set is significantly different in many characters. It is most likely that the mutational load was caused by long-lasting asexual reproduction, manifested by the accumulation of deleterious aberrations in two original genomes at different intensities. Seemingly, *A. tenuicollis* might be the ideal candidate for an ancestral sparring partner for *A. huronensis* concerning interspecific hybridization [7]. The sexually reproducing diploid *A. tenuicollis* forms a genetically closest cluster with *A. huronensis* [9]. These two species are morphologically similar and occur in China [7, 11, 34]. On the other hand, the last congener *A. sagittatus* originated from other parts of Asia (the Amur River basin, Caspian Sea Drainage, and Japan), and its genetic affinity with *A. huronensis* is a bit weaker [9, 36, 42]. Hopefully, detailed cytogenetic research of sexual *Atractolytocestus* spp. as well as the Chinese *A. huronensis* population will solve the problem.

## Conclusions

Detailed cytogenetic analyses have provided a deeper understanding of the triploid nature and hypothetical origin of the invasive carp tapeworm *A. huronensis*. However, no final conclusion can be made concerning the origin of the ancient polyploidization event. Solving this mystery requires further cytogenetic research of the recently discovered Chinese populations of *A. huronensis*, as well as of both congeners, which might have served as sexual progenitors. Moreover, further molecular studies of *A. huronensis* triploid lineages could help us to better understand which deleterious effects of the long-term absence of sex need to be countered. Another unsolved problem concerns the function of minimized telomeres in parthenogenetic *A. huronensis*. The future comprehensive comparison of closely related asexual and sexual congeneric species therefore remains a vital part of concerted efforts to solve the paradox of invasive nature and abandoned sexuality.

## Abbreviations

ANOVA: analysis of variance
AT: adenine, thymine
C-banding: centromere banding stain
DABCO: 1,4-diazabicyclo[2.2.2]octane
DAPI: 4′,6-diamino-2-phenylindole
FISH: fluorescent *in situ* hybridization
ITSs: interstitial telomeric sequences
NOR: nucleolar organization region
PBS: phosphate-buffered saline
PCR: polymerase chain reaction
rDNA: ribosomal deoxyribonucleic acid
SSC: saline-sodium citrate buffer
2q cen: post-centromeric locus
2q int: intercalar locus
